# Predictive model of suicide risk in Colombian university students: quantitative analysis of associated factors

**DOI:** 10.3389/fpsyt.2024.1291299

**Published:** 2024-05-24

**Authors:** Sandra Constanza Cañón Buitrago, Juan Manuel Pérez Agudelo, Mariela Narváez Marín, Olga Lucia Montoya Hurtado, Gloria Isabel Bermúdez Jaimes

**Affiliations:** ^1^ Medical Research Group - Medicine Program - Faculty of Health Sciences, University of Manizales, Manizales, Caldas, Colombia; ^2^ Clinical Psychology and Health Processes Group, Psychology Program, Faculty of Social and Human Sciences, Manizales University, Manizales, Caldas, Colombia; ^3^ Human Abilities, Health, and Inclusion Group - Physiotherapy - Research Department, Colombian School of Rehabilitation, Manizales, Caldas, Colombia; ^4^ Human Abilities, Health, and Inclusion Group - Research Department, Colombian School of Rehabilitation, Manizales, Caldas, Colombia

**Keywords:** suicide, suicide attempt, predictive model, university students, risk factors

## Abstract

**Introduction:**

The risk of suicide and completed suicides among young university students presents critical challenges to mental and public health in Colombia and worldwide. Employing a quantifiable approach to comprehend the factors associated with these challenges can aid in visualizing the path towards anticipating and controlling this phenomenon.

**Objective:**

Develop a predictive model for suicidal behavior in university students, utilizing predictive analytics.

**Method:**

We conducted an observational, retrospective, cross-sectional, and analytical research study at the University of Manizales, with a focus on predictive applicability. Data from 2,436 undergraduate students were obtained from the research initiative “Building the Future: World Mental Health Surveys International College Students.”

**Results:**

The top ten predictor variables that generated the highest scores (ranking coefficients) for the sum of factors were as follows: history of sexual abuse (13.21), family history of suicide (11.68), medication (8.39), type of student (7.4), origin other than Manizales (5.86), exposure to cannabis (4.27), exposure to alcohol (4.42), history of physical abuse (3.53), religiosity (2.9), and having someone in the family who makes you feel important (3.09).

**Discussion:**

Suicide involves complex factors within psychiatric, medical, and societal contexts. Integrated detection and intervention systems involving individuals, families, and governments are crucial for addressing these factors. Universities also play a role in promoting coping strategies and raising awareness of risks. The predictive accuracy of over 80% in identifying suicide risk underscores its significance.

**Conclusion:**

The risk factors related to suicidal behavior align with the findings in specialized literature and research in the field. Identifying variables with higher predictive value enables us to take appropriate actions for detecting cases and designing and implementing prevention strategies.

## 1 Introduction

In 2017, as part of the Sustainable Development Goals, there was a reevaluation of the global mental health agenda aimed at expanding services for individuals affected by mental disorders (1). This reassessment drew upon more than a decade of research evidence emphasizing interdisciplinary practices across various contexts, with an emphasis on preventing and treating mental disorders and promoting mental health. Despite significant progress in research, the practical impact on everyday life has unfortunately been slow. Mental health services often lag behind physical health services in terms of quality. The collective failure to address this crisis results in a loss of human potential and unnecessary suffering (2). Moreover, suicide rates have been steadily increasing year after year, with one out of every hundred deaths globally attributed to this cause (3).

In the first five months of 2023, Colombia reported 11,411 cases of suicide, with the majority occurring in males (9,933). This represents an increase compared to the same period in 2022, which saw a total of 11,055 cases, with 9,564 involving males. Among these cases, 51.9% (5,920) occurred in the age range of 20 to 39 years, and 17 cases were reported in Manizales (4). According to a report from the Public Health Department of Manizales, there were 30 cases of suicide in 2021, four fewer than in 2020. Consistent with a global trend, the majority of these cases (93%) were among males (5).

The rise in suicidal behavior among individuals aged 15 to 44 years is concerning, particularly within the age range of university students. In recent years, this demographic has faced the complex challenge of suicide, necessitating comprehensive intervention (6). Thus, it is imperative to identify the risk factors that render this population more vulnerable and work towards prevention.

Numerous research studies have pinpointed multiple risk factors associated with suicidal ideation, suicide attempts, and completed suicides. These factors encompass family disintegration, changes in residence particularly relocating away from home to attend university (7) the frequency of suicide attempts, and family history of suicide attempts and completed suicides (8–10). The authors underscore the significance of recognizing that a family history of suicidal behavior may lead to a form of learning through imitation. They emphasize that while suicidal behavior itself is not hereditary, there exists a genetic predisposition to certain mental disorders, such as depression, which in turn serves as a significant risk factor associated with this behavior.

It’s crucial to verify students’ health background and family history because within the realm of mental disorders and substance misuse, suicidal tendencies can pose particularly challenging obstacles. The complexity of these conditions and their profound impact on individuals’ emotional, cognitive, and psychological well-being underscore the need for thorough consideration (11). Depression, for instance, correlates with heightened risks of suicidal thoughts and actions, alongside other conditions like anxiety, bipolar disorder, and schizophrenia. Substance dependence, or addiction, further heightens these risks by fostering emotional instability and impairing judgment (12). Additionally, sedentary lifestyles, often associated with these disorders, can exacerbate both physical and mental health issues, thereby amplifying the risk of suicide. Physical inactivity may contribute to obesity, diabetes, and various chronic ailments, exacerbating emotional distress and psychological vulnerability (11, 13). Addressing these health challenges necessitates specialized support to encompass both mental and physical well-being, thereby mitigating the risk of suicidal tendencies through tailored therapies and continuous monitoring by mental health professionals.

The correlation between depression, feelings of hopelessness, and suicide attempts has been extensively documented by various researchers in their studies (14, 15). Furthermore, factors such as the dissolution of romantic relationships and intolerance towards diverse sexual orientations have also been linked to suicide attempts, unfortunately serving as grounds for discrimination (7, 16). Family history has similarly been identified as a risk factor (15, 17). In Mexican university students who reported suicidal ideation, associated factors comprised growing up with only the mother during childhood, alcohol, or drug consumption, expressing negative or ambivalent thoughts about oneself, and perceiving an uncertain future (18).

Studies conducted on Colombian populations have identified predictors of suicide attempts, including experiences of physical abuse and maltreatment, as well as suicidal ideation (19). Depression and feelings of hopelessness have also emerged as significant predictors (9, 15). Particularly among young and adolescent individuals who identify as homosexual, the factor most strongly associated with suicidal ideation was having experienced sexual abuse (20).

Family relationships and bonds exert a significant influence on suicidal behaviors. When these interactions are marked by persistent difficulties and imbalances, they can escalate into risk factors for a family member attempting suicide (21). Conversely, strong, and healthy family bonds characterized by cohesion and effective communication act as protective factors for adolescents expressing a desire to live (22, 23).

The early identification of these protective factors within specific populations is a valuable tool for contributing to the prevention of suicidal behaviors. Consequently, the utilization of data analytics has become indispensable for generating predictive models that can enhance the customization of prevention strategies and early interventions to the unique realities of each studied population. In this regard, predictive algorithm models are employed (24), which, when applied to the context of suicide, can aid in identifying individuals at a higher risk of engaging in such behavior.

In this context, a systematic review conducted in 2019 analyzed a total of 7,306 research studies to assess the accuracy of predictive suicide models. Among these studies, 17 cohort studies met the inclusion criteria, representing a collection of 64 unique prediction models across five countries, with participation from over 14 million individuals. The overall classification accuracy of these models was generally good, exceeding 0.80 in most cases. However, the predictive validity related to a positive outcome for suicide mortality was extremely low, with values of less than 0.01 in most instances (25).

In Mexico, a study was conducted with 911 high school students to develop a predictive model for suicidal ideation. The study utilized multiple regression analysis, considering variables such as family support, depression, and adjustment issues within the school environment. Their findings revealed that depression was the most closely associated factor with suicidal ideation. Nevertheless, the study underscores the significance of acknowledging that there are multiple factors that can precipitate this condition. Therefore, they advocate for the implementation of strategies that address these variables both within the family and the school environments (14).

In the university context, it is crucial to understand and distinguish between intentional self-harm and suicide attempts, as although both behaviors may involve self-inflicted physical harm, their underlying motivations and objectives are different. Intentional self-harm, such as cutting or burning the skin, is typically done as a way to relieve emotional distress or cope with overwhelming stress and emotions. Often, these actions are not intended to cause death but rather to provide temporary relief from emotional tension. On the other hand, suicide attempts involve a conscious and deliberate desire to end one’s own life. These behaviors are motivated by deep despair, hopelessness, or psychological suffering, and pose a significant risk to the individual’s life. It is essential to recognize and address these differences in order to provide appropriate intervention and necessary support to university students facing emotional and mental challenges (26).

Suicide is a profoundly complex event, characterized by a multitude of interacting factors that form an inseparable whole (23). In this context, it is crucial to acknowledge that various components, spanning biological, emotional, psychological, and social aspects, intricately interact to shape the human experience within society. This encompasses sociological, economic, historical, and religious dimensions.

Recognizing the risk factors for suicide within university populations holds immense value for educational institutions. This knowledge empowers them to provide support to their students, gain insights into their mental well-being, and develop effective prevention strategies. Therefore, this research aims to construct a predictive model for suicidal behavior among university students.

## 2 Method

We conducted an observational, retrospective, cross-sectional, and analytical research study using a predictive-applicative approach. The data were sourced from the database compiled as part of the research initiative “Building the Future: World Mental Health Surveys International College Students”. Our sample consisted of 2,436 students from the University of Manizales, who participated voluntarily after providing informed consent. The research’s characteristics and procedures were thoroughly reviewed and approved by the Ethics Committee of the University of Manizales.

### 2.1 Data treatment and analysis plan

The questionnaire was online and the information was collected in a census of the student population of the University of Manizales, summoning them in person to computer rooms to accompany them in any questions that arose. The research project utilized information categorized into independent and dependent variables. The data underwent preprocessing using the open-access online platform OpenRefine (27). The consolidated database underwent both descriptive and analytical procedures utilizing the IBM-licensed SPSS (Statistical Package for the Social Sciences) version 24. The proposed methodology began with univariate descriptives, followed by multivariate procedures rooted in discriminant analysis (Fisher’s Linear Discriminant Functions) (28, 29).

The data utilization/data science pyramid (30) was initially considered for operationalizing variables individually and in their multiple associations, progressing towards the proposed predictive model based on discriminant or classification procedures for the categorical variable of interest (dependent variable): suicide attempt within the last year. Overall descriptive evaluation relied on presenting absolute and relative frequencies. For the exploratory relational level of qualitative variables, symmetric and asymmetric contingency tables were generated, incorporating proportions, frequencies, and totals.

The choice of discriminant analysis as the proposed predictive model for the research process was based on the following principles:

- Inherent discriminant (dependency) capacity sought in the research: the ability to classify suicide attempts based on nominal or numerical participant characteristics.
- Presence of categorical and/or numerical independent variables.
- Inclusion of cases outnumbered variables (at least by a factor of 10x) in the model (n = 2436; variables = 40).
- Confirmatory capacity for new cases added to the model.
- Attainment of a linear combination of independent variables maximizing the distinction of a membership group.
- Estimation or probability metric of classifying each particular case into an interest group.
- Absence of collinearity or multicollinearity among independent variables.
- Presence of intragroup homogeneity and extra group heterogeneity.

A metric of 0.7 (70.0%) was considered as the lower limit to imply adequate classification for the model.

### 2.2 Predictive analytical route

The predictive analytical route with multivariate mediation, discriminant, and under Fisher coefficient parameters creates a model for group membership classification. This model allows, through discriminant functions, the classification into two or more groups with categorical definition. For the investigative process, it was fundamental to orient the prediction towards one of two possible categories: with suicidal risk or without suicidal risk (dependent variable). The discriminant model generates linear combinations of variables (predictors - independents) for establishing the classification (28, 29). The variables included in the predictive model were taken from the macro project “Building the Future: World Mental Health Surveys International College Students,” research that preliminarily sought to establish the descriptive bases of the central phenomenon of analysis, which subsequently allowed the creation of the model proposal.

The information processing was carried out with the aim of reaching high levels of data cleansing; however, the intention of this phase to complement multivariate (predictive) data analytics did not consider participant selection or inclusion/exclusion criteria due to the retrospective nature and grounding in secondary research data. The data available for the analysis process were not declared as restricted in the criteria included in the informed consent. On the other hand, the exclusion of participant identification variables or data (anonymization for the analysis process) was critical for the model implementation.

The research did not consider subdividing the data sets for predictive model metrics, especially pertinent to machine learning or Artificial Intelligence models. Multivariate analysis through classification functions (discriminant) has certain characteristics: its accuracy improves with more variables and cases, and its mutually exclusive nature allows clear categorization. However, subdividing groups for multivariate analytics testing in this case could increase the risk of type II errors, potentially accepting the null hypothesis. The discriminant model using Fisher’s linear functions doesn’t include modeling graphics; instead, graphical results focus on descriptive elements. The model’s main contribution lies in the discrimination coefficients table, which is included as part of the results.

**Table 1** variables considered for the research process.

**Table 1:** Independent variables associated with the predictive model.

Variable	Categories
University Program	1. Business Administration
	2. Social Communication and Journalism
	3. Public Accounting
	4. Law
	5. Floating
	6. Systems and Telecommunications Engineering
	7. Medicine
	8. National and International Marketing
	9. Psychology
	10. Bachelor’s in Primary Education with an English Emphasis
Semester	1 to 12
Type of Student	1. New - 2. Returning
Origin Different from Manizales	1. Yes, from another municipality or city in the same department
	2. Yes, from another department
	3. Yes, from another country
	4. I am from this municipality or city
Origin	1. Caldas
	2. Other departments or countries
Prevalence of Emotional Disorder	1. Yes
	2. No
Age	
Gender	1. Male
	2. Female
	3. Transgender (male to female)
	4. Transgender (female to male)
Sexual Orientation	1. Heterosexual
	2. Gay or Lesbian
	3. Bisexual
	4. Asexual
	5. Not sure
Perception of Physical Health	1. Excellent 2. Very good 3. Good 4. Fair 5. Poor
Perception of Mental Health	1. Excellent 2. Very good 3. Good 4. Fair 5. Poor
Violence between Parents	1. Very often 2. Often 3. Sometimes 4. Very few times 5. Never
Family History of Mental Health Problems	1. Very often 2. Often 3. Sometimes 4. Very few times 5. Never
Family History of Alcohol Problems	1. Very often 2. Often 3. Sometimes 4. Very few times 5. Never
Family History of Suicide	1. Very often 2. Often 3. Sometimes 4. Very few times 5. Never
History of Physical Abuse	1. Very often 2. Often 3. Sometimes 4. Very few times 5. Never
History of Emotional Abuse	1. Very often 2. Often 3. Sometimes 4. Very few times 5. Never
History of Sexual Abuse	1. Very often 2. Often 3. Sometimes 4. Very few times 5. Never
Light Exercise	1. Every day or almost every day
	2. 3-4 days a week
	3. 1-2 days a week
	4. Less than 1 day a week
	5. Never
Moderate Exercise	1. Every day or almost every day
	2. 3-4 days a week
	3. 1-2 days a week
	4. Less than 1 day a week
	5. Never
Balanced Meals	1. Every day or almost every day
	2. 3-4 days a week
	3. 1-2 days a week
	4. Less than 1 day a week
	5. Never
Gets 8 Hours of Sleep	1. Every day or almost every day
	2. 3-4 days a week
	3. 1-2 days a week
	4. Less than 1 day a week
	5. Never
Consumes Fruits and Vegetables	1. Every day or almost every day
	2. 3-4 days a week
	3. 1-2 days a week
	4. Less than 1 day a week
	5. Never
Psychological Help Seeking	1. Yes - 2. No
Medication	1. Yes - 2. No
Alcohol Consumption	1. Yes - 2. No
Cannabis (Marijuana) Consumption	1. Yes - 2. No
Cocaine Consumption	1. Yes - 2. No
Other Psychoactive Substance Consumption	1. Yes - 2. No
Mixed Substance Consumption (MCO)	1. Yes - 2. No
Medication Consumption	1. Yes - 2. No
Family Member Makes You Feel Important	1. Very often 2. Often 3. Sometimes 4. Very few times 5. Never
Feel Loved and Cared for by Family	1. Very often 2. Often 3. Sometimes 4. Very few times 5. Never
Emotionally Close to Family Members	1. Very often 2. Often 3. Sometimes 4. Very few times 5. Never
Religion	1. Christianity/ Catholicism 2. Judaism 3. Islam 4. Atheism 5. Agnosticism 6. Other religion
Religious or Spiritual Help Seeking	1. Very often 2. Often 3. Sometimes 4. Very few times

### 2.3 Ethical considerations

Due to the confidential nature of the information, approval was sought from the institution. It was clarified that since data from the mental health field were being analyzed, it was unnecessary to include personal details such as names, identification, addresses, etc. The Ethics Committee of the University of Manizales provided its endorsement, supported by resolution SUI1020. This approval ensured that the research adhered to ethical standards and upheld participant privacy and rights.

## 3 Results

A total of 2,436 records were included in the multivariate model using discriminant analysis, with 98% of cases (n = 2,388) weighted as non-suicidal attempts in the last year. The multivariate analysis applied for predicting the occurrence of suicide attempts in the last year was conducted using a path of multiple regression analysis with linear Fisher discriminant functions. Binary classification coefficients were generated for the predictor variables of interest, achieving a correct classification rate of 87.4% for the predicted event (refer to **Table 2**).

**Table 2:** Rank function coefficients for suicide attempt in the past year.

Variables	Suicide Attempt	
	Yes	No
Program	1,026	1,044
Semester	-0.433	-0.330
Type of Student	7.123	7.677
Different Origin from Manizales	5.933	5.795
Origin	-6.815	-5.734
Prevalence of Emotional Disorder	-2.215	-1.939
Age	0.792	0.815
Gender	0.291	0.337
Sexual Orientation	0.095	0.118
Perception of Physical Health	0.682	0.378
Perception of Mental Health	0.083	1.183
Violence between Parents	0.027	-0.444
Family History of Mental Health Problems	-0.251	0.129
Family History of Alcohol Problems	0.491	0.714
Family History of Suicide	11.899	11.452
History of Physical Abuse	3.517	3.543
History of Emotional Abuse	-1.541	-0.146
History of Sexual Abuse	12.855	13.564
Light Exercise	0.777	0.885
Moderate Exercise	-0.233	-0.122
Balanced Meals	0.389	0.557
Gets 8 Hours of Sleep	1.253	1.369
Consumes Fruits and Vegetables Three Times Daily	0.446	0.212
Psychological Help Seeking	-0.072	0.562
Medication	7.852	8.920
Alcohol Consumption	4.380	4.465
Cannabis Consumption	4.908	3.622
Cocaine Consumption	0.820	-1.899
Other Psychoactive Substance Consumption	0.933	0.708
Medication Consumption	-0.178	0.742
Family Member Makes You Feel Important	3.124	3.046
Feel Loved and Cared for by Family	0.892	0.931
Emotionally Close to Family Members	-0.192	-0.073
Family Cohesion	-2.248	-2.222
Religion	1.817	1.659
Religiosity	3.173	2.624
Frequency of Attending Religious Services or Events	0.175	0.030
Considers Oneself Spiritual	0.523	0.547
Religious or Spiritual Help Seeking	-0.320	-0.057
Constant (β)	-83.223	-93.536

The top 10 predictor variables based on their score for the sum of factors were: History of sexual abuse (13.21), family history of suicide (11.68), medication (8.39), type of student (7.4), different origin than Manizales (5.86), exposure to cannabis (4.27), exposure to alcohol (4.42), history of physical abuse (3.53), being religious (2.9), and someone in the family makes you feel important (3.09). The five variables that subtracted from the phenomenon of interest were: origin (-6.27), family cohesion (-2.23), prevalence of emotional disorder (-2.08), history of emotional abuse (-0.84), and academic semester (-0.38).

Discriminant coefficients facilitate the generation of classifications for the event of interest. As an illustration, the prediction for the theoretical case of a new student in systems engineering is provided (refer to **Table 3**).

**Table 3:** Application of classification coefficients by integrating variables associated with a case.

Variables	Suicide Attempt		Caso		Suicide Attempt	
	Yes	No	Value^*^	Category	with intent	no attempt
Program	1.026	1.044	5	Engineering	5.13	5.22
Semester	0.433	0.330	1	First semester	-0.43	-0.33
Type of Student	7.123	7.677	0	New	0.00	0.00
Different Origin from Manizales	5.933	5.795	2	Yes	11.87	11.59
Origin	6.815	5.734	1	Caldas	-6.81	-5.73
Prevalence of Emotional Disorder	2.215	1.939	1	No	-2.22	-1.94
Age	0.792	0.815	25	25 years	19.81	20.37
Gender	0.291	0.337	3	Male	0.87	1.01
Sexual Orientation	0.095	0.118	4	Heterosexual	0.38	0.47
Perception of Physical Health	0.682	0.378	2	Good	1.36	0.76
Perception of Mental Health	0.083	1.183	3	Very Good	0.25	3.55
Violence between Parents	0.027	0.444	3	Very Few Times	0.08	-1.33
Family History of Mental Health Problems	0.251	0.129	4	Never	-1.01	0.52
Family History of Alcohol Problems	0.491	0.714	4	Never	1.96	2.85
Family History of Suicide	11.899	11.452	3	Very Few Times	35.70	34.36
History of Physical Abuse	3.517	3.543	3	Very Few Times	10.55	10.63
History of Emotional Abuse	1.541	0.146	3	Very Few Times	-4.62	-0.44
History of Sexual Abuse	12.855	13.564	4	Never	51.42	54.25
Light Exercise	0.777	0.885	1	Less than 1 day a week	0.78	0.89
Moderate Exercise	0.233	0.122	2	1 to 2 days a week	-0.47	-0.24
Balanced Meals	0.389	0.557	3	3 to 4 days a week	1.17	1.67
Gets 8 Hours of Sleep	1.253	1.369	1	Less than 1 day a week	1.25	1.37
Consumes Fruits and Vegetables 3 Times Daily	0.446	0.212	1	Less than 1 day a week	0.45	0.21
Psychological Help Seeking	0.072	0.562	1	No	-0.07	0.56
Medication	7.852	8.920	1	No	7.85	8.92
Alcohol Consumption	4.380	4.465	1	Yes	4.38	4.47
Cannabis Consumption	4.908	3.622	0	No	0.00	0.00
Cocaine Consumption	0.820	1.899	0	No	0.00	0.00
Other Psychoactive Substance Consumption	0.933	0.708	0	No	0.00	0.00
Medication Consumption	0.178	0.742	0	No	0.00	0.00
Family Member Makes You Feel Important	3.124	3.046	4	Very Often	12.50	12.18
Feel Loved and Cared for by Family	0.892	0.931	4	Very Often	3.57	3.72
Emotional Bond with Family Members	0.192	0.073	4	Very Often	-0.77	-0.29
Family Cohesion	2.248	2.222	4	Very Often	-8.99	-8.89
Religion	1.817	1.659	0	Christianity/Catholicism	0.00	0.00
Religious Practice	3.173	2.624	2	Regular	6.35	5.25
Frequency of Attending Religious Services or Events	0.175	0.030	2	1 to 3 times a month	0.35	0.06
Considers Oneself Spiritual	0.523	0.547	3	Much	1.57	1.64
Religious or Spiritual Help Seeking	0.320	0.057	4	Always	-1.28	-0.23
Constant (β)	-83.223	-93.536	1	-	-83.22	-93.54
				Sum of Coefficients	69.70	73.57

*The value assigned to the category corresponds to the number coded in the SPSS program for each value of the nominal or ordinal variable.

The summation of coefficient products yields a value for each category of the dependent variable of interest (suicide attempt in the last year). The value of 73.57 in the “No attempt” column surpasses the sum of values in the “Attempt” column, leading to the classification of the case as “No suicide attempt.” With the discriminant capacity of the test, 9 out of 10 cases are correctly classified in each category (87.4% correct classification rate).

## 4 Discussion

The issue of suicide encompasses a multitude of factors spanning various contexts, including psychiatric, medical, biological, psychological, familial, educational, social, and environmental dimensions. These factors may be preventable in certain instances, manageable in others, and potentially eradicated through the concerted efforts of individuals, families, social institutions, and governmental bodies, facilitated by an integrated system of detection and intervention. Within the specific context of universities, the transition into this environment coupled with the inherent changes of adolescence presents a significant challenge for young individuals. They must adapt to a new setting that can significantly impact their lifestyle, potentially necessitating leaving behind their familial home and relocating to a different city, all while adjusting to the unique educational strategies of higher education. Inadequately managing these transitions renders them more susceptible to various situations that could affect their mental health, including suicide (31). According to a study by Nock et al. (2013) conducted across 21 countries, including Colombia, mental disorders are prevalent among university students and often manifest even before enrollment (32). It is common to encounter a sense of vulnerability among foreign students who leave their home and city, along with their established support networks, to confront an entirely unfamiliar and sometimes threatening environment. Drawing upon the interpersonal theory of suicide, this sense of non-belonging, loneliness, and subjective lack of positive relationships experienced by these students could lead them to perceive suicide as a viable solution (33). Feelings of abandonment, detachment from family, and social isolation were identified by Larrobla et al. (2017) as key triggering factors for suicidal behavior (15). Conversely, living in a communicative and supportive familial and community environment, where rights and feelings are respected, serves as a protective factor (30). Being from a different origin than Manizales was found to be associated with suicidal ideation among surveyed students.

Moreover, the university environment encompasses not only physical dimensions but also social, academic, and particularly relational aspects, each carrying personal significance for individuals. This significance directly impacts their mental well-being and their ability to navigate daily challenges. However, many students lack effective coping mechanisms to manage these new demands, leading each to construct their own responses based on the meanings they attribute to their experiences and their evaluations of their environment’s relevance (31).

In a study conducted by Lara et al. (2015) involving Mexican university students, it was found that positive aspects of the university environment have a beneficial effect on students’ perceptions of their quality of life (18). Adolescence represents a stage of life where skills acquired from earlier phases are utilized and further developed as individuals establish new interactions within family and social spheres. During this time, youth seek a sense of belonging by affiliating with various groups while also striving for independence and solidifying their identity (32). Presently, young individuals are confronted with new challenges for which many are ill-prepared. These challenges, compounded by factors such as substance abuse, alcohol consumption, unprotected sexual activity, poor dietary habits, and a sedentary lifestyle, render them more susceptible to psychosocial vulnerabilities (33).

Having a positive self-concept has been recognized as a significant protective factor, as it fosters feelings of self-contentment that contribute to overall well-being (34). Furthermore, the influence of interpersonal bonds on individual development cannot be understated, as they play a pivotal role in shaping emotional, social, and cognitive aspects (35). This becomes particularly crucial during adolescence, a period marked by unique challenges where young individuals necessitate an environment that provides them with the necessary tools for navigating such obstacles. When family cohesion is lacking, it can escalate into a risk factor by depriving its members of the affection, protection, and security they require. Adolescents require validation and support from within the family unit. In our research, the absence of family support exhibited a positive correlation with suicidal ideation. Students from a university in Valledupar reported struggles in communicating with their parents due to perceived emotional disconnection, resulting in episodes of anxiety, depression, and feelings of isolation that adversely affect both their academic performance and emotional well-being (36). The lack of family support has been associated with an increased likelihood of suicidal ideation, underscoring the imperative need to address this issue proactively.

Furthermore, maltreatment is often found in families with low cohesion, a factor that leaves lasting consequences that can manifest during adolescence in various ways such as depression, post-traumatic stress, suicide attempts, and substance use (37). In our investigation, a history of maltreatment was one of the predictive factors for suicidal behaviors. Timely detection of such cases among students allows for appropriate intervention to reinforce coping strategies for trauma.

Beyond emotional support, families play a pivotal role in emotion regulation, which is closely linked to social skills. Adolescents with effective coping styles for problem-solving are less likely to experience suicidal ideation (35). Conversely, lacking social skills can lead to alternative behaviors as coping mechanisms, including substance use. Substance use, particularly cannabis and alcohol consumption, as well as medication, has been identified as one of the most relevant factors for suicidal ideation and attempts (36–39). These findings were also observed in our study.

The Colombian Family Welfare Institute (ICBF, 2018) asserts that preventive measures have proven effective in suicide prevention. Harmonious family relationships, a positive school environment, balanced health status, and an optimistic view of the future, combined with a commitment to education, can help neutralize adverse factors (40).

Teachers play a central role in achieving comprehensive student management, requiring appropriate knowledge and the development of necessary skills. A health-promoting university is one that integrates strengthening human development and the quality of life for its students and staff into its institutional project. In this sense, it undertakes actions aimed at providing stimuli, security, and satisfaction to promote self-care and contribute to reducing the risks they face (41).

It’s evident that health isn’t an isolated factor in an individual’s life. It’s the result of various elements: genetics, the environment, and the neurobiological effects of experiences during sensitive stages, such as adolescence (42). Health is built in daily life and within different contexts where interactions occur. It’s also a responsibility both on an individual and societal level, with universities playing a crucial role. Therefore, educational processes should encompass health education, the detection of risk factors, and the reinforcement of coping strategies that empower students to choose options that contribute to their well-being, health, and improved quality of life.

This is supported by a study conducted in Korea in 2018 with 291 college students, where the findings suggest a model of suicidal ideation focusing on protective factors and a positive outlook. This study aimed to develop a theoretical model that explains suicidal ideation in college students and incorporated factors such as resilience, sense of belonging, adjustment to college life, depression, and stress. Structural equation modeling was used to test the hypotheses, finding that a sense of belonging and depression had significant direct effects, while college stress and a sense of belonging had significant indirect effects. A sense of belonging had the greatest influence, with an explanatory power of 37% for suicidal ideation in students (43).

Timely detection and evaluation of suicide risk are essential, as suicide can often be preventable. Effective measures can be taken based on this evaluation to manage these risks efficiently.

Koppmann (2020) found that individuals who died by suicide had consulted healthcare professionals and had risk factors such as untreated psychiatric illness, severe stress, and previous suicide attempts. Previous suicide attempts have been considered one of the strongest predictors of a suicidal act, highlighting their value in preventive approaches (44).

Our study found that variables such as history of sexual abuse, family history of suicide, and medication, among others, are significant predictors of suicide risk. This highlights the importance of identifying such factors to design effective preventive strategies. We found studies addressing the risk of suicide among university student populations in the past five years. These studies share a common focus on identifying and predicting suicide risk, but they differ in their methods and specific findings.

The study “One-year Incidence, Predictors, and Accuracy of Prediction of Suicidal Thoughts and Behaviors from the First to Second Year of University” examines the incidence of suicidal thoughts and behaviors among university students during their first year. It uses a longitudinal approach to evaluate the predictive accuracy of a multivariate risk model. Although both studies share the goal of preventing suicide among university students, they differ in their methodological approach and specific predictors considered (45).

Another study, “Prediction of Suicidal Ideation among Chinese College Students based on Radial Basis Function Neural Network,” focuses on developing a predictive model for suicidal ideation among Chinese students using neural networks. This study highlights the importance of considering psychological and behavioral factors, such as sleep quality and previous symptoms of suicidal ideation, as key predictors (46).

Finally, the study “Psychosocial Factors and Clinical Predictors of Suicide Risk in College Students” stands out by exploring the psychosocial and clinical factors associated with suicide risk among Colombian university students. It uses a variety of assessment scales, such as the Plutchik Suicide Risk Scale and the Barratt Impulsiveness Scale, to identify significant predictors of suicide risk (47).

It is believed that universities, within their functions, should not only provide students with tools to develop their cognitive abilities for their future professional performance but also ensure comprehensive education where the human factor is an essential component for professionals to harness their capacities and contribute to society (48–50).

## 5 Conclusion

The analysis of 2,436 records using multivariate discriminant analysis has provided valuable insights into the predictors of suicidal behavior among university students. This comprehensive approach yielded significant findings, including a commendable correct classification rate of 87.4% for predicting suicide attempts within the last year using multiple regression analysis with linear Fisher discriminant functions.

Among the top 10 predictor variables identified were a history of sexual abuse, family history of suicide, medication, type of student, and exposure to substances such as cannabis and alcohol. These findings underscore the multifaceted nature of suicidal behavior. Conversely, variables like origin, family cohesion, and prevalence of emotional disorders displayed negative associations with the phenomenon of interest.

The discriminant coefficients derived from the analysis facilitated accurate classifications. For instance, in a theoretical case of a new student in systems engineering, the model correctly classified 9 out of 10 cases in each category, highlighting its efficacy in identifying individuals at risk of suicidal behavior.

Addressing the identified risk factors is crucial in developing targeted intervention strategies. Factors such as a history of sexual abuse, family history of suicide, and substance abuse underscore the need for comprehensive mental health support systems within university settings. Additionally, negative associations with variables like family cohesion underscore the importance of promoting supportive familial relationships to mitigate the risk of suicidal behavior among students.

Overall, this study significantly contributes to our understanding of suicidal behavior among university students and emphasizes the necessity for proactive measures to address mental health concerns within educational institutions. Further research and implementation of targeted interventions based on the identified predictors are warranted to effectively reduce the incidence of suicide attempts among this vulnerable population.

The results of this initiative integrate previous research efforts supporting risk factors for suicidal behavior alongside the analytical capacity of multivariate analysis with predictive purposes for suicide risk. A predictive result exceeding 80% in risk identification cannot go unnoticed, making it a clear starting point for advancing and anticipating the phenomenon of suicide. This research introduces a unique metric that should be considered in the evaluation and monitoring of individuals with impaired quality of life in the field of mental health, especially in environments marked by high anxiety, stress, and depression such as the university setting.

The modeling of sociodemographic, clinical characterization, lifestyle, and psychological variables represents an innovative effort to guide cost-effective anticipation in the face of a specific challenge of this millennium: the mental health of the university population.

The study boasts strengths such as analyzing a wide sample of 2,436 records using multivariate discriminant analysis, providing a robust foundation for understanding suicidal behavior among university students, and achieving an impressive correct classification rate of 87.4%. Additionally, it identifies key risk factors such as history of sexual abuse, family history of suicide, and substance use, which carry significant practical implications for targeted intervention strategies. However, it does have limitations in terms of generalizability and potential omitted variables, highlighting the need for external validations and careful consideration when interpreting the results for future research and mental health policy developments in university settings.

## Data availability statement

The raw data supporting the conclusions of this article will be made available by the authors, without undue reservation.

## Ethics statement

The studies involving humans were approved by Comité de Bioética de la Universidad de Manizales. The studies were conducted in accordance with the local legislation and institutional requirements. The participants provided their written informed consent to participate in this study. Written informed consent was obtained from the individual(s) for the publication of any potentially identifiable images or data included in this article.

## Author contributions

MM: Conceptualization, Funding acquisition, Investigation, Resources, Writing – original draft, Writing – review & editing. SB: Conceptualization, Funding acquisition, Investigation, Resources, Writing – original draft, Writing – review & editing. JA: Data curation, Methodology, Writing – review & editing. OH: Funding acquisition, Investigation, Project administration, Supervision, Writing – original draft. GJ: Conceptualization, Funding acquisition, Resources, Writing – original draft.
